# Multidisciplinary clinics in cancer—models, metrics, and meaning: a review

**DOI:** 10.1093/oncolo/oyag208

**Published:** 2026-06-04

**Authors:** Seohyuk Lee, Jim W Doolin, David E Avigan, Stefan Balan, Peter Chang, Kristen Crowell, Martin Dib, Kelly Downey, Charly Edmiston, Panagiotis Fidias, Jason A Freed, Leslie A Garrett, Lauren Hertan, Scharukh Jalisi, David McDermott, Evangelos Messaris, Umut Sarpel, Ammar Sarwar, Meghan Shea, Rafael Vega, Peter D Whooley, Jennifer L Wilson, Elizabeth A Mittendorf, Jessica A Zerillo

**Affiliations:** Department of Medicine, Beth Israel Deaconess Medical Center, Boston, MA 02215, United States; Department of Hematology and Medical Oncology, Lahey Hospital and Medical Center, Burlington, MA 01805, United States; Division of Hematology and Hematologic Malignancies, Beth Israel Deaconess Medical Center, Boston, MA 02215, United States; Hematology and Medical Oncology, Winchester Hospital, Winchester, MA 01890, United States; Department of Urology, Beth Israel Deaconess Medical Center, Boston, MA 02215, United States; Department of Surgery, Beth Israel Deaconess Medical Center, Boston, MA 02215, United States; Department of Surgery, Beth Israel Deaconess Medical Center, Boston, MA 02215, United States; Division of Medical Oncology, Beth Israel Deaconess Medical Center, Boston, MA 02215, United States; Department of Medicine, Beth Israel Deaconess Medical Center, Boston, MA 02215, United States; Hematology and Medical Oncology, Exeter Hospital, Exeter, NH 03833, United States; Division of Hematology and Hematologic Malignancies, Beth Israel Deaconess Medical Center, Boston, MA 02215, United States; Division of Gynecologic Oncology, Beth Israel Deaconess Medical Center, Boston, MA 02215, United States; Department of Radiation Oncology, Beth Israel Deaconess Medical Center, Boston, MA 02215, United States; Department of Otolaryngology, Beth Israel Deaconess Medical Center, Boston, MA 02215, United States; Division of Medical Oncology, Beth Israel Deaconess Medical Center, Boston, MA 02215, United States; Department of Surgery, Beth Israel Deaconess Medical Center, Boston, MA 02215, United States; Department of Surgery, Beth Israel Deaconess Medical Center, Boston, MA 02215, United States; Division of Interventional Radiology, Beth Israel Deaconess Medical Center, Boston, MA 02215, United States; Division of Medical Oncology, Beth Israel Deaconess Medical Center, Boston, MA 02215, United States; Department of Surgery, Beth Israel Deaconess Medical Center, Boston, MA 02215, United States; Division of Medical Oncology, Beth Israel Deaconess Medical Center, Boston, MA 02215, United States; Department of Surgery, Beth Israel Deaconess Medical Center, Boston, MA 02215, United States; Division of Breast Surgery, Beth Israel Deaconess Medical Center, Boston, MA 02215, United States; Division of Medical Oncology, Beth Israel Deaconess Medical Center, Boston, MA 02215, United States

**Keywords:** cancer, multidisciplinary clinic, models, outcomes

## Abstract

Few studies have been published on the existing models of cancer multidisciplinary clinics (MDCs) and no studies have conclusively compared the implications of different MDC models. We aimed to characterize MDC structural elements and reported quality measures through a narrative review of cancer MDCs in the United States. Forty-one unique MDCs were examined (8 breast, 13 gastrointestinal [GI], 8 genitourinary [GU], 4 head and neck [HN], 3 lung, 5 other cancers). MDCs were most commonly weekly (19/41) and evaluated new patients (9/41). All breast and most GU (5/8) and lung (2/3) MDCs were asynchronous whereas all HN MDCs were synchronous. Interdisciplinary discussions most frequently preceded provider visits, except for HN and other cancer MDCs. Medical, radiation, and surgical oncology were almost always included across all MDCs. The 41 unique institutional MDCs were reported across 54 studies (10 breast, 17 GI, 13 GU, 4 HN, 4 lung, 6 other cancers). Outcome measures were investigated by 38/54 (70%) studies, of which 13 reported overall survival and 6 (46%) noted a statistically significant improvement with MDCs. All lung and approximately half of GI (7/17), breast (5/10), and HN (2/4) MDCs examined a process measure. Structure and balance measures were not as widely reported. Current understanding of MDCs is based on disparate reports with significant heterogeneity in MDC structures and reported outcomes. Further investigation is needed to better elucidate the impact of MDCs in cancer care outcomes across different cancer diagnoses.

Implications for Practice Current understanding of cancer multidisciplinary clinics (MDCs) is based on disparate reports with significant heterogeneity in MDC structures and reported outcomes. Further investigation is needed to better elucidate the impact of MDCs in cancer care outcomes across different cancer diagnoses.

## Introduction

Quality cancer care, as underscored by an American Society of Clinical Oncology (ASCO) and European Society for Medical Oncology (ESMO) consensus statement, is often rooted in the adoption of a multidisciplinary approach.^1^ Various models of multidisciplinary cancer care have been implemented across different practice settings.^2^^,^^3^ In traditional tumor boards, the patient’s provider typically formulates the treatment plan following consultative multidisciplinary discussion without the patient present. Multidisciplinary clinics (MDCs) offer a more inclusive approach, with specialties—such as medical oncology (MO), radiation oncology (RO), and surgery—meeting and coordinating a treatment plan within the same patient encounter.^4^

Cancer management is rapidly evolving with new therapeutic modalities, omission of therapies to minimize toxicity, and changing therapy sequencing. Given these dynamic treatment options, patients’ active participation is integral in developing a patient-centered treatment plan. There is other potential value to MDCs besides reaching the best clinical decision with incorporation of patients’ individual needs. If coordination of care within an MDC can reduce delays in initial access, improve accuracy and timing of decision-making, shorten time to treatment initiation, and improve patient satisfaction, there are likely clinical benefits to patients, enhanced professional gratification for providers, and financial value for the institution.^5–7^ Despite these potential benefits, few studies have examined the models of cancer MDCs and no studies have compared the implications of different MDC models. Similarly, there is a paucity of literature on if and how cancer MDCs may be tailored to most effectively deliver care to patients with different cancer types and ultimately how they may impact outcomes.

We aimed to provide a narrative review to summarize the structural elements and quality measures associated with different models of cancer MDCs in the United States (U.S.). The focus of this review is those studies which have explicitly described the structures of their respective MDCs and met our aforementioned criteria. Of note, a recent meta-analysis^8^ has suggested that same-day MDCs may improve outcomes without detriments to patient satisfaction or financial costs however the specific clinic structures and features that confer benefit remain unclear. Our review adds to this recent work in describing MDC models and metrics to allow more cross-study comparisons and reproducibility of the specific MDC models that may be most effective for different cancer diagnoses.

## Methods

### Search strategy

PubMed was queried from inception to July 2024 using a search strategy containing keywords related to cancer and MDCs (see Supplemental Material). One study personnel (S.L.) screened 11920 citations by title and abstract and, when relevant, full-text to identify U.S.-based MDCs meeting our definition of MDCs as same-day in-person or telehealth clinical encounters with a patient diagnosed with cancer conducted by physicians of ≥2 different specialties. These criteria reflect the definition of optimal multidisciplinary care by the National Comprehensive Cancer Control Program as collaborative, prospective, and synchronous.^9^ All studies that explicitly described their MDC structures and could be determined to match our definition were included. International studies were excluded given the different health care insurance and reimbursement structures, heterogeneous care delivery models, and potentially different patient, provider, and institutional expectations for optimal multi-disciplinary care outside of the U.S. We additionally searched ASCO Annual Meeting and Quality Care Symposium abstractions through October 2024; none described MDCs structures.

### Analysis

Based on previously identified MDC models,^3^ MDCs were classified as operating either synchronously (if patients met with all providers simultaneously) or asynchronously (if provider visits were separate but occurring same-day). Determination of MDC structures (synchronous versus asynchronous; in-person versus telehealth setting) and timing of interdisciplinary case reviews (ICRs) relative to patient-facing evaluations were based on reported manuscript descriptions. ICR timing was classified as either preceding or following the provider visit, or unknown. Visit setting was defined as in-person or telehealth (video- or phone-visit). Studies were examined for reporting of four primary healthcare quality measure types as outlined by the Agency for Healthcare Research and Quality and the Institute for Healthcare Improvement^10^^,^^11^: (1) structure (eg, clinical volume); (2) process, representing concordance of received care with expected care (eg, time-to-treatment); (3) outcome, signifying care impact on the patient (eg, mortality); and (4) balance (eg, use of non-indicated care).

## Observations

### Included studies

Fifty-four studies of MDCs (10 breast,^5^^,^^12–20^ 17 gastrointestinal (GI),^21–37^ 13 genitourinary (GU),^38–50^ 4 head and neck (HN),^51–54^ 4 lung,^55–58^ 6 other cancers^59–64^) met our inclusion criteria, among which 41 unique institutional MDCs were examined. The 41 unique institutional MDCs included: 8 breast, 13 gastrointestinal [GI], 8 genitourinary [GU], 4 head and neck [HN], 3 lung, 5 other cancers.

Among breast cancer studies, 8 institutional breast MDCs were examined across 9 of these studies, with the remaining 1 study reflecting analyses of population-based data and thus evaluated only for reported measures and not MDC model characteristics. Remaining studies across all other cancer types were based on institutional MDCs, examining 13 GI MDCs (5 colorectal, 3 each hepatobiliary and pancreas, 2 various GI cancers), 8 GU MDCs (6 prostate, 1 each bladder and all GU cancers), 4 HN MDCs, 3 lung MDCs, and 5 other cancer MDCs (2 various cancers and 1 each hematologic, neurologic, skin cancers).

### Model summary

**Figure 1.**
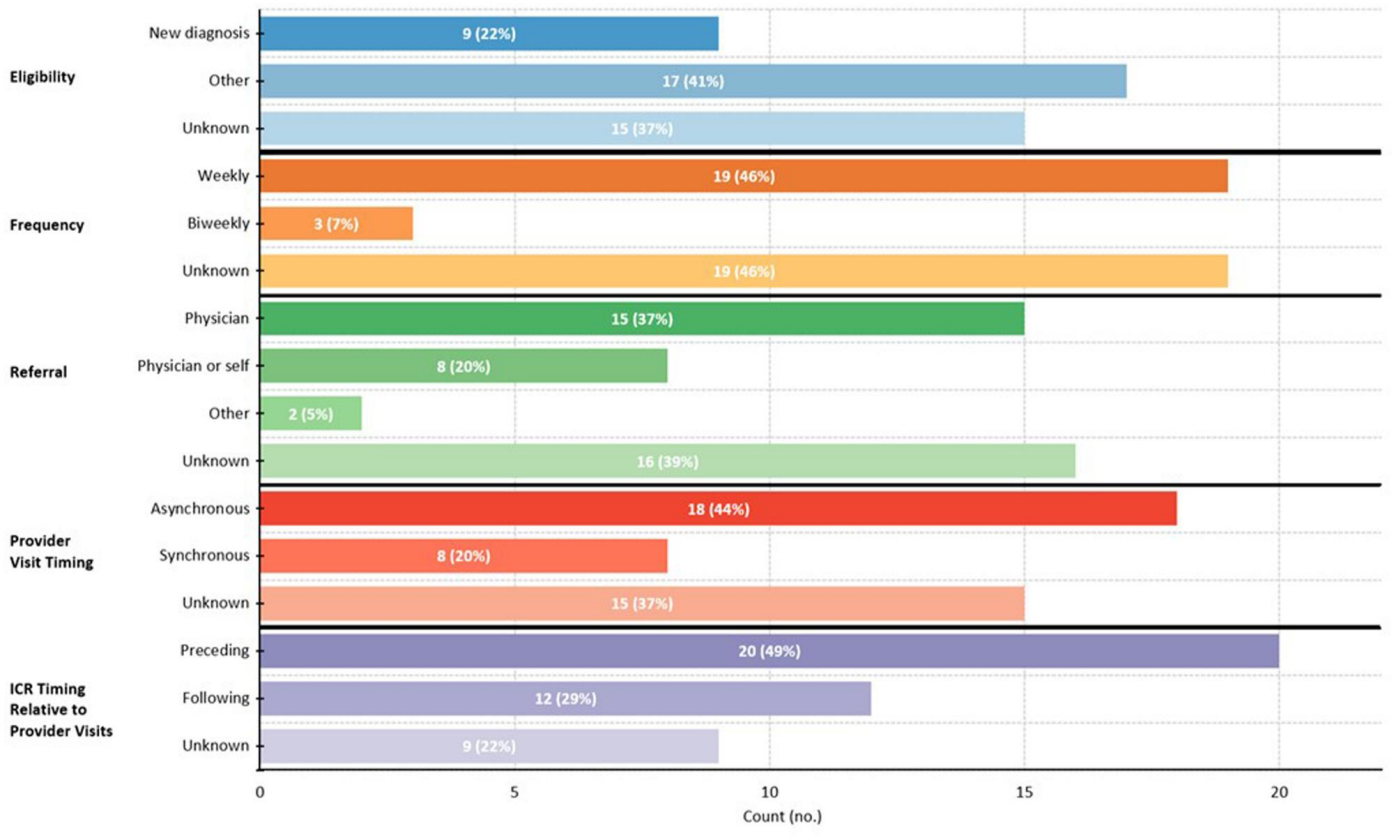
Multidisciplinary cancer clinic characteristics overall. Within-bar values represent count numbers and percent proportions relative to all 41 examined institutional MDCs. Abbreviations: GI = gastrointestinal; GU = genitourinary; H&N = head and neck; ICR = interdisciplinary case review; MDC = multidisciplinary clinic.

**Figure 2.**
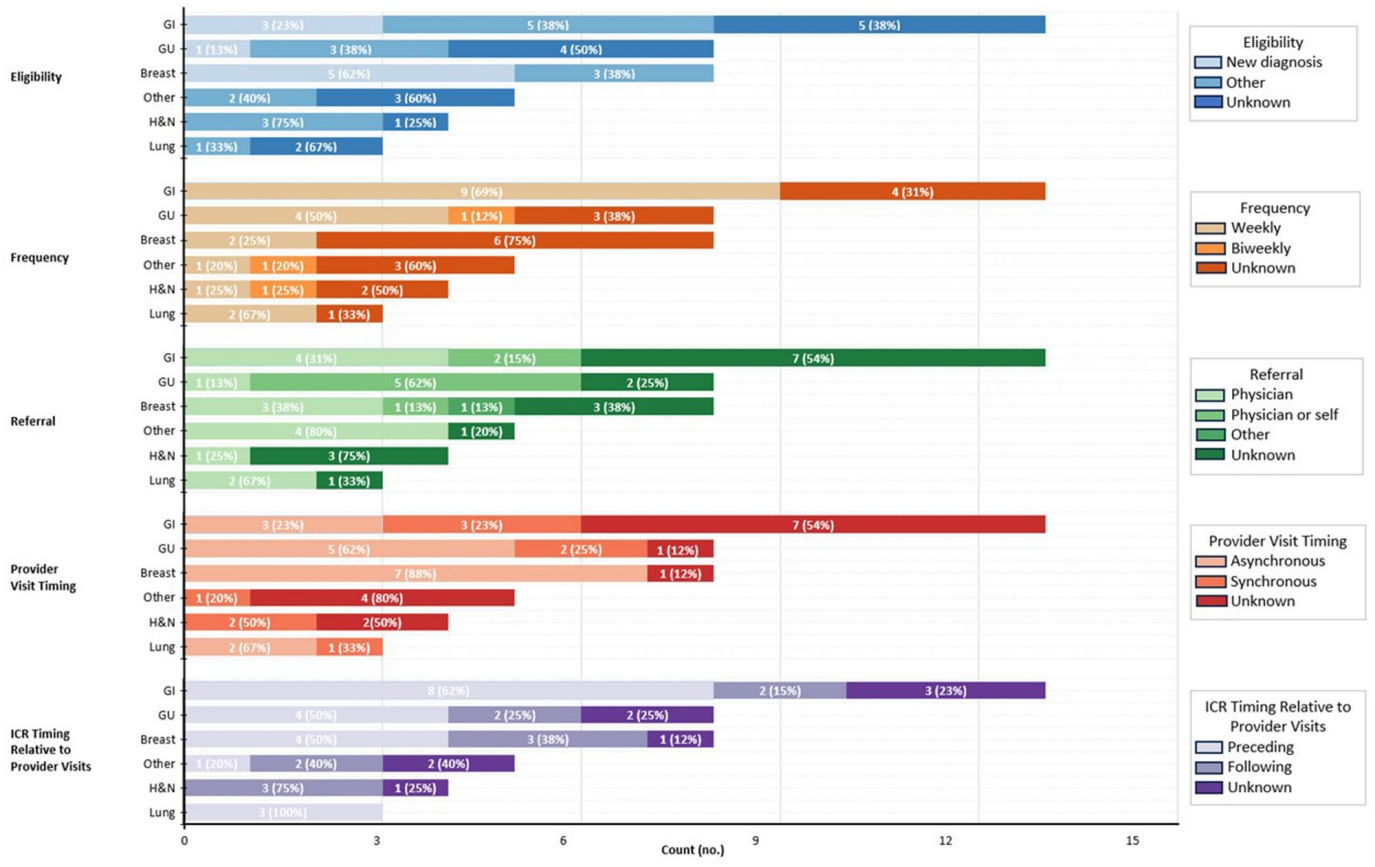
Multidisciplinary cancer clinic characteristics by cancer diagnosis. Within-bar values represent count numbers and percent proportions relative to total number of examined MDCs for that cancer diagnosis (13 GI, 8 GU, 8 Breast, 4 H&N, 3 Lung, 5 Other). Abbreviations: GI = gastrointestinal; GU = genitourinary; H&N = head and neck; ICR = interdisciplinary case review; MDC = multidisciplinary clinic.

**Figure 3.**
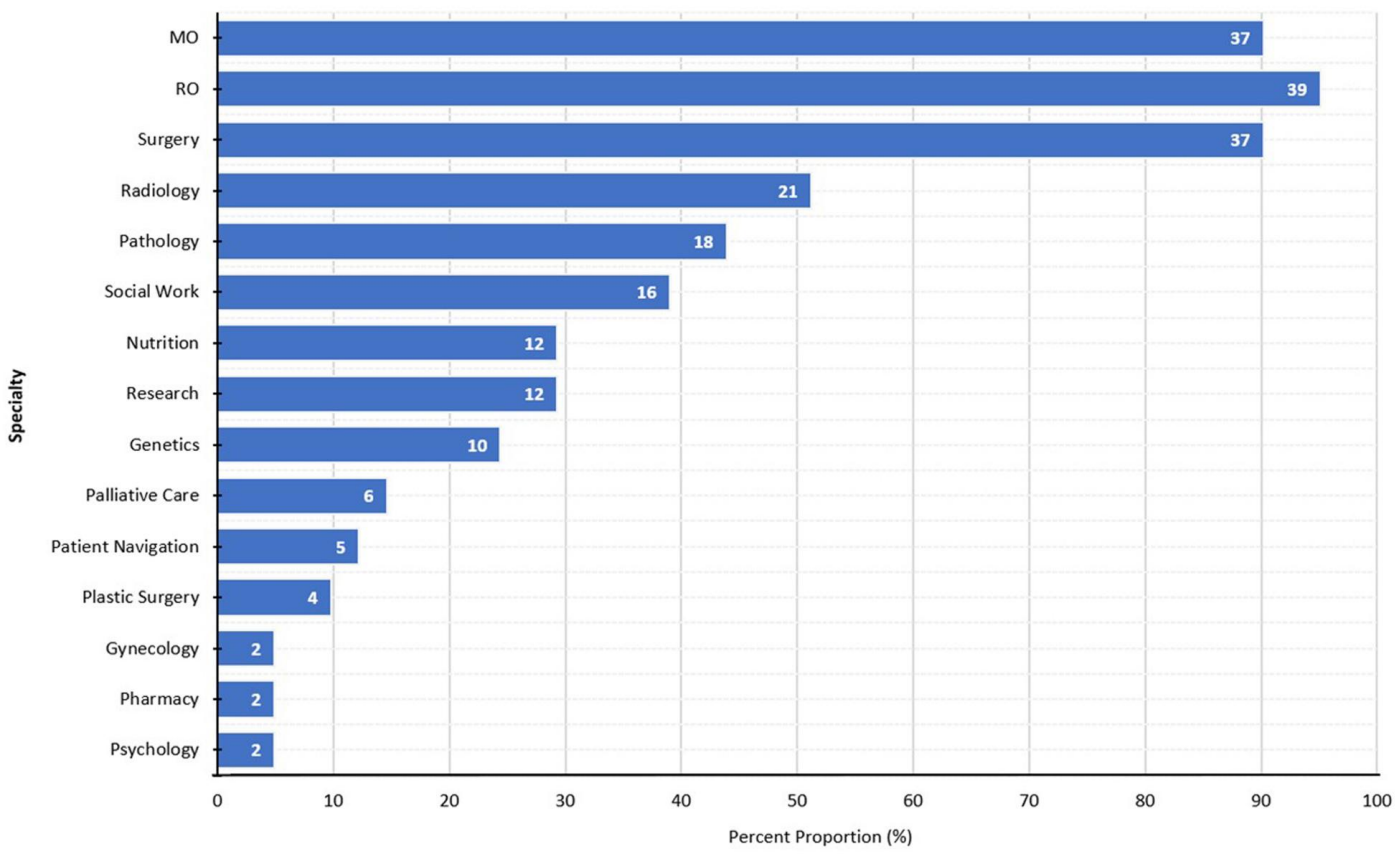
Multidisciplinary cancer clinic specialty involvement across all cancer diagnoses. Only specialties that were involved in MDCs of ≥2 cancer diagnoses are represented. Percent proportion values are relative to all 41 examined institutional MDCs. Within-bar values represent count values. Abbreviations: MO = medical oncology; RO = radiation oncology.

**Figure 4.**
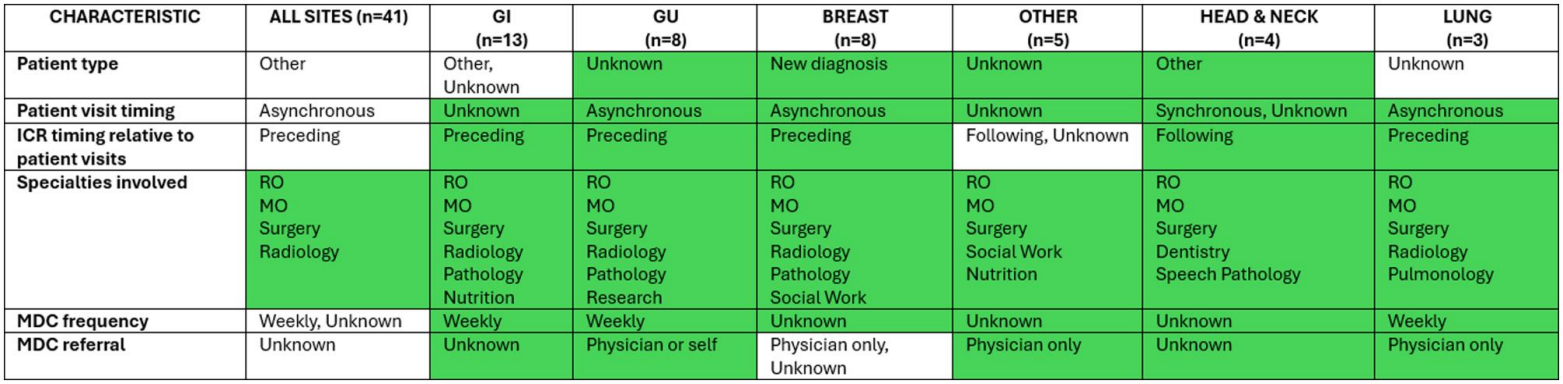
Most common characteristics of MDCs overall and by cancer diagnosis. Multiple entries for a characteristic indicate a tie for most common, except for “Specialties Involved” in which all specialties involved in ≥50% of included MDCs are listed. Green highlight denotes applicable to ≥50% of included MDCs. Abbreviations: GI = gastrointestinal; GU = genitourinary; ICR = interdisciplinary case review; MDC = multidisciplinary clinic; MO = medical oncology; RO = radiation oncology.

Forty-one individual MDCs were included in the examination of MDC models (Table 1, Figures 1 and 2). MDC referral was most frequently by a physician, and many relied on clinical staff to obtain and review records prior to the MDC visit. Most common criterion of patient eligibility to be evaluated in an MDC was a new cancer diagnosis at 22% (9/41). All breast and most GU (5/8) and lung (2/3) MDCs operated in an asynchronous manner whereas all HN MDCs operated synchronously. MDCs were most commonly weekly, except for HN and other cancers which had similar rates of weekly and biweekly occurrences. In contrast to most cancer diagnoses, HN (3/4) and other cancer (2/5) MDCs were structured with ICR following, not preceding, provider visits. When described, all ICRs were separate from patient-facing assessments. In-person, rather than telehealth, MDCs were most common across all cancer diagnoses. MO, RO, and surgery were almost always included across MDCs of all cancer diagnoses. Radiology and pathology were frequently included for GI, GU, and breast MDCs, with the former additionally common in lung MDCs (Figure 3). For approximately half of GI MDCs, there was involvement by genetics and nutrition, whereas GU MDCs frequently included research personnel and breast MDCs often involved social work (SW). Figure 4 illustrates a high-level summary of the most common characteristics overall and by cancer diagnosis.

**Table 1.** Summary of characteristics of multidisciplinary cancer clinics reported in the literature overall and by cancer diagnosis.

		All cancers (*n* = 41)	GI					GU (*n* = 8)	Breast (*n* = 8)	Other (*n* = 5)	Head & neck (*n* = 4)	Lung (*n* = 3)
			Total GI (*n* = 13)	Colorectal (*n* = 5)	Pancreas (*n* = 3)	HPB (*n* = 3)	Various GI (*n* = 2)					
**Patient type, no. (%)**	*Other*	17 (41.5)	5 (38.5)	1 (20.0)	0 (0)	3 (100)	1 (50.0)	3 (37.5)	3 (37.5)	2 (40.0)	3 (75.0)	1 (33.3)
	*Unknown*	15 (36.6)	5 (38.5)	3 (60.0)	1 (33.3)	0 (0)	1 (50.0)	4 (50.0)	0 (0)	3 (60.0)	1 (25.0)	2 (66.7)
	*New diagnosis*	9 (22.0)	3 (23.1)	1 (20.0)	2 (66.7)	0 (0)	0 (0)	1 (12.5)	5 (62.5)	0 (0)	0 (0)	0 (0)
**Specialties involved, no. (%)**	*RO*	39 (95.1)	13 (100)	5 (100)	3 (100)	3 (100)	2 (100)	8 (100)	8 (100)	3 (60.0)	4 (100)	3 (100)
	*MO*	37 (90.2)	13 (100)	5 (100)	3 (100)	3 (100)	2 (100)	6 (75.0)	8 (100)	4 (80.0)	3 (75.0)	3 (100)
	*Surgery*	37 (90.2)	13 (100)	5 (100)	3 (100)	3 (100)	2 (100)	8 (100)	7 (87.5)	3 (60.0)	4 (100)	2 (66.7)
	*Radiology*	21 (51.2)	9 (69.2)	3 (60.0)	1 (33.3)	3 (100)	2 (100)	4 (50.0)	4 (50.0)	1 (20.0)	1 (25.0)	2 (66.7)
	*Pathology*	18 (43.9)	7 (53.8)	3 (60.0)	1 (33.3)	2 (66.7)	1 (50.0)	4 (50.0)	4 (50.0)	1 (20.0)	1 (25.0)	1 (33.3)
	*Social Work*	16 (39.0)	5 (38.5)	2 (40.0)	2 (66.7)	0 (0)	1 (50.0)	2 (25.0)	4 (50.0)	3 (60.0)	1 (25.0)	1 (33.3)
	*Nutrition*	12 (29.3)	7 (53.8)	3 (60.0)	3 (100)	0 (0)	1 (50.0)	0 (0)	0 (0)	3 (60.0)	1 (25.0)	1 (33.3)
	*Research*	12 (29.3)	4 (30.8)	1 (20.0)	3 (100)	0 (0)	0 (0)	6 (75.0)	1 (12.5)	0 (0)	1 (25.0)	0 (0)
	*Genetics*	10 (24.4)	6 (46.2)	4 (80.0)	1 (33.3)	0 (0)	1 (50.0)	2 (25.0)	2 (25.0)	0 (0)	0 (0)	0 (0)
	*Palliative Care*	6 (14.6)	4 (30.8)	0 (0)	2 (66.7)	2 (66.7)	0 (0)	0 (0)	0 (0)	0 (0)	1 (25.0)	1 (33.3)
	*Patient Navigation*	5 (12.2)	1 (7.7)	1 (20.0)	0 (0)	0 (0)	0 (0)	1 (12.5)	2 (25.0)	0 (0)	1 (25.0)	0 (0)
	*Plastic Surgery*	4 (9.8)	0 (0)	0 (0)	0 (0)	0 (0)	0 (0)	0 (0)	3 (37.5)	1 (20.0)	0 (0)	0 (0)
	*Gynecology*	2 (4.9)	1 (7.7)	1 (20.0)	0 (0)	0 (0)	0 (0)	0 (0)	0 (0)	1 (20.0)	0 (0)	0 (0)
	*Pharmacy*	2 (4.9)	0 (0)	0 (0)	0 (0)	0 (0)	0 (0)	0 (0)	0 (0)	1 (20.0)	0 (0)	1 (33.3)
	*Psychology*	2 (4.9)	0 (0)	0 (0)	1 (33.3)	0 (0)	0 (0)	1 (12.5)	1 (12.5)	0 (0)	0 (0)	0 (0)
	*Other*	–	Gastroenterology (5, 38.5); Interventional Radiology (4, 30.8); Hepatology (3, 23.1); Transplant Surgery (2, 15.4); Enterostomal Therapy (1, 7.7); Hepatobiliary Surgery (1, 7.7); Primary Care (1, 7.7); Thoracic Surgery (1, 7.7)	Gastroenterology (2, 40.0) Enterostomal Therapy (1, 20.0); Hepatobiliary Surgery (1, 20.0); Primary Care (1, 20.0); Thoracic Surgery (1, 20.0)	Gastroenterology (1, 33.3)	Hepatology (3, 100); Interventional Radiology (3, 100); Transplant Surgery (1, 33.3)	Gastroenterology (2, 100); Interventional Radiology (1, 50.0); Transplant Surgery (1, 50.0)	Financial Counseling (1, 12.5)	Lymphedema Therapy (1, 12.5)	Geriatrics (2, 40.0); Bone Marrow Transplant (1, 20.0); Dermatology (1, 20.0); Infectious Disease (1, 20.0); Ophthalmology (1, 20.0); Otorhinolaryngology (1, 20.0); Physical/Occupational Therapy (1, 20.0);	Dentistry (2, 50.0); Speech Pathology (2, 50.0); Audiology (1, 25.0)	Pulmonology (2, 66.7)
**MDC frequency, no. (%)**	*Unknown*	19 (46.3)	4 (30.8)	2 (40.0)	0 (0)	1 (33.3)	1 (50.0)	3 (37.5)	6 (75.0)	3 (60.0)	2 (50.0)	1 (33.3)
	*Weekly*	19 (46.3)	9 (69.2)	3 (60.0)	3 (100)	2 (66.7)	1 (50.0)	4 (50.0)	2 (25.0)	1 (20.0)	1 (25.0)	2 (66.7)
	*Biweekly*	3 (7.3)	0 (0)	0 (0)	0 (0)	0 (0)	0 (0)	1 (12.5)	0 (0)	1 (20.0)	1 (25.0)	0 (0)
**MDC referral, no. (%)**	*Unknown*	16 (39.0)	7 (53.8)	3 (60.0)	1 (33.3)	1 (33.3)	2 (100)	2 (25.0)	3 (37.5)	1 (20.0)	3 (75.0)	1 (33.3)
	*Physician*	15 (36.6)	4 (30.8)	2 (40.0)	1 (33.3)	1 (33.3)	0 (0)	1 (12.5)	3 (37.5)	4 (80.0)	1 (25.0)	2 (66.7)
	*Physician or self*	8 (19.5)	2 (15.4)	0 (0)	1 (33.3)	1 (33.3)	0 (0)	5 (62.5)	1 (12.5)	0 (0)	0 (0)	0 (0)
	*Other*	2 (4.9)	0 (0)	0 (0)	0 (0)	0 (0)	0 (0)	0 (0)	1 (12.5)	0 (0)	0 (0)	0 (0)
**Setting, no. (%)**	*In-Person*	40 (97.6)	12 (92.3)	4 (80.0)	3 (100)	3 (100)	2 (100)	8 (100)	8 (100)	5 (100)	4 (100)	3 (100)
	*Telehealth*	2 (4.9)	1 (7.7)	1 (20.0)	0 (0)	0 (0)	0 (0)	0 (0)	1 (12.5)	0 (0)	0 (0)	0 (0)
**Patient visit timing, no. (%)**	*Asynchronous*	18 (43.9)	3 (23.1)	2 (40.0)	1 (33.3)	0 (0)	0 (0)	5 (62.5)	7 (87.5)	0 (0)	0 (0)	2 (66.7)
	*Unknown*	15 (36.6)	7 (53.8)	2 (40.0)	1 (33.3)	3 (100)	1 (50.0)	1 (12.5)	1 (12.5)	4 (80.0)	2 (50.0)	0 (0)
	*Synchronous*	8 (19.5)	3 (23.1)	1 (20.0)	1 (33.3)	0 (0)	1 (50.0)	2 (25.0)	0 (0)	1 (20.0)	2 (50.0)	1 (33.3)
**ICR timing relative to patient visits, no. (%)**	*Preceding*	20 (48.8)	8 (61.5)	2 (40.0)	2 (66.7)	3 (100)	1 (50.0)	4 (50.0)	4 (50.0)	1 (20.0)	0 (0)	3 (100)
	*Following*	12 (29.3)	2 (15.4)	1 (20.0)	0 (0)	0 (0)	0 (0)	2 (25.0)	3 (37.5)	2 (40.0)	3 (75.0)	0 (0)
	*Unknown*	9 (22.0)	3 (23.1)	2 (40.0)	1 (33.3)	0 (0)	1 (50.0)	2 (25.0)	1 (12.5)	2 (40.0)	1 (25.0)	0 (0)

Abbreviations: GI = gastrointestinal; GU = genitourinary; HPB = hepatobiliary; ICR = interdisciplinary case review; MDC = multidisciplinary clinic; MO = medical oncology; RO = radiation oncology; SO = surgical oncology.

### Metrics summary

Table 2 summarizes measures reported in the 54 included studies by categories of structure, process, outcome, and balance. Outcome measures—such as changes in diagnostic interpretations or treatment recommendations, survival, and patient satisfaction—were the most reported, investigated by at least half of all studies for each cancer diagnosis. Thirteen studies^15^^,^^30–33^^,^^36^^,^^39^^,^^40^^,^^48^^,^^52^^,^^55^^,^^56^^,^^58^ reported overall survival, among which 6^30^^,^^33^^,^^39^^,^^40^^,^^48^^,^^55^ (46%) noted a statistically significant improvement with MDCs. All lung and approximately half of GI (7/17), breast (5/10), and HN (2/4) MDC studies examined a process measure—namely time-to-treatment initiation—whereas this was less common in other cancer diagnoses. Structure measures were not as widely reported, although nearly half of GU MDC studies and a quarter of studies evaluating HN and lung MDCs did so. Balance measures were the least commonly investigated, approximately 11% (6/54) of all studies.

**Table 2.** Summary of measures associated with multidisciplinary cancer clinics reported in the literature overall and by cancer diagnosis.

Measure type	All cancers (*n* = 54)	GI					GU (*n* = 13)	Breast (*n* = 10)	Other (*n* = 6)	Head & neck (*n* = 4)	Lung (*n* = 4)
		Total GI (*n* = 17)	Pancreas (*n* = 7)	Colorectal (*n* = 5)	HPB (*n* = 3)	Various GI (*n* = 2)					
***Structure, total, no. (%)***	**12 (22.2)**	**2 (11.8)**	**0 (0)**	**1 (20.0)**	**1 (33.3)**	**0 (0)**	**6 (46.2)**	**1 (10.0)**	**1 (16.7)**	**1 (25.0)**	**1 (25.0)**
**Patient retention, no. (%)**	7 (13.0)	2 (11.8)	0 (0)	1 (20.0)	1 (33.3)	0 (0)	4 (30.8)	0 (0)	0 (0)	1 (25.0)	0 (0)
**Patient volume, no. (%)**	8 (14.8)	1 (20.0)	0 (0)	1 (20.0)	0 (0)	0 (0)	6 (46.2)	1 (10.0)	0 (0)	0 (0)	0 (0)
**Financial costs, no. (%)**	2 (3.7)	0 (0)	0 (0)	0 (0)	0 (0)	0 (0)	0 (0)	0 (0)	1 (16.7)	0 (0)	1 (25.0)
***Process, total, no. (%)***	**18 (33.3)**	**7 (41.2)**	**2 (28.6)**	**3 (60.0)**	**2 (66.7)**	**0 (0)**	**0 (0)**	**5 (50.0)**	**0 (0)**	**2 (50.0)**	**4 (100)**
**Time to treatment initiation, no. (%)**	17 (31.5)	6 (35.3)	2 (28.6)	3 (60.0)	1 (33.3)	0 (0)	0 (0)	5 (50.0)	0 (0)	2 (50.0)	4 (100)
**Time to oncologic assessment, no. (%)**	6 (11.1)	3 (17.6)	0 (0)	2 (40.0)	1 (33.3)	0 (0)	0 (0)	1 (10.0)	0 (0)	1 (25.0)	1 (25.0)
***Outcome, total, no. (%)***	**38 (70.4)**	**12 (70.6)**	**6 (85.7)**	**2 (40.0)**	**3 (100)**	**1 (50.0)**	**10 (76.9)**	**7 (70.0)**	**4 (66.7)**	**2 (50.0)**	**3 (75.0)**
**Survival, no. (%)**	14 (25.9)	5 (29.4)	4 (57.1)	0 (0)	1 (33.3)	0 (0)	3 (23.1)	1 (10.0)	1 (16.7)	1 (25.0)	3 (75.0)
**Receipt of treatment type, no. (%)**	13 (24.1)	7 (41.2)	3 (42.9)	2 (40.0)	2 (66.7)	0 (0)	3 (23.1)	2 (20.0)	0 (0)	1 (25.0)	0 (0)
**Clinical trial enrollment, no. (%)**	10 (18.5)	5 (29.4)	3 (42.9)	0 (0)	2 (66.7)	0 (0)	3 (23.1)	0 (0)	0 (0)	1 (25.0)	1 (25.0)
**Change in treatment recommendation, no. (%)**	8 (14.8)	4 (23.5)	1 (14.3)	0 (0)	2 (66.7)	1 (50.0)	1 (7.7)	2 (20.0)	1 (16.7)	0 (0)	0 (0)
**Change in diagnostic interpretation, no. (%)**	7 (13.0)	4 (23.5)	1 (14.3)	0 (0)	2 (66.7)	1 (50.0)	1 (7.7)	2 (20.0)	0 (0)	0 (0)	0 (0)
**Patient satisfaction, no. (%)**	7 (13.0)	0 (0)	0 (0)	0 (0)	0 (0)	0 (0)	3 (23.1)	2 (20.0)	2 (33.3)	0 (0)	0 (0)
**Receipt of diagnostic type, no. (%)**	3 (5.6)	1 (5.9)	0 (0)	1 (20.0)	0 (0)	0 (0)	0 (0)	1 (10.0)	0 (0)	0 (0)	1 (25.0)
**Receipt of specialty referral or evaluation, no. (%)**	5 (9.3)	2 (11.8)	1 (14.3)	1 (20.0)	0 (0)	0 (0)	0 (0)	1 (10.0)	0 (0)	2 (50.0)	0 (0)
***Balance, total, no. (%)***	**6 (11.1)**	**2 (11.8)**	**1 (14.3)**	**0 (0)**	**1 (33.3)**	**0 (0)**	**2 (15.4)**	**0 (0)**	**0 (0)**	**0 (0)**	**2 (50.0)**
**Receipt of non-indicated diagnostic, no. (%)**	1 (1.9)	0 (0)	0 (0)	0 (0)	0 (0)	0 (0)	1 (7.7)	0 (0)	0 (0)	0 (0)	0 (0)
**Resource utilization prior to treatment initiation, no. (%)**	5 (9.3)	2 (11.8)	1 (14.3)	0 (0)	1 (33.3)	0 (0)	1 (7.7)	0 (0)	0 (0)	0 (0)	2 (50.0)

Abbreviations: GI = gastrointestinal; GU = genitourinary; HPB = hepatobiliary.

#### Breast cancer

All included breast MDCs operated asynchronously involving consecutive but separate provider visits with each relevant specialty however sequencing varied with some MDCs starting with ICR followed by provider visits^12^^,^^17^^,^^19^^,^^20^ and others inversely.^5^^,^^13^^,^^16^. One center began with patient evaluations by the surgical team followed by ICR then consecutive visits with the relevant specialties.^20^ Similarly, another center involved patient evaluations by a physician and advanced practice provider followed by ICR during which consensus recommendations were formulated.^16^ Three MDCs incorporated scheduling of additional necessary diagnostics within the clinic.^13^^,^^19^^,^^20^

Specialists from MO, RO, and surgery were involved in all but one of the breast MDCs which excluded surgery. Four breast MDCs had representatives from SW, pathology, and radiology; three involved plastic surgery; and two each included genetics and patient navigators. Two breast MDCs were conducted weekly; the frequency of the remaining six were not described. Eligibility criteria for six breast MDCs included patients with newly diagnosed breast cancer, although one included additional specifications such as molecular subtype and stage. One center only included older adults with stage pT1 and c/pN0 hormone receptor-positive invasive cancer who had already undergone breast-conserving surgery,^12^ and another center included only those patients with an established breast cancer diagnosis who were presenting for second opinion.^20^ One site evaluated all patients with newly diagnosed breast cancer,^19^ whereas another had nurse navigators actively screen for and identify appropriate patients based on prespecified clinical criteria related to hormone receptor status and lymph node involvement.^17^

Process and outcome measures were the most investigated breast MDC endpoints by five^12^^,^^13^^,^^15^^,^^17^^,^^19^ and seven^5^^,^^12^^,^^15^^,^^16^^,^^18–20^ of the ten studies, respectively. Time-to-treatment was evaluated by 50% of studies, although there were variations in how the baseline (eg, diagnosis, initial MDC visit) and treatment (eg, neoadjuvant chemotherapy, surgery) timepoints were defined. Diagnostic interpretation changes, treatment recommendation changes, treatment type receipt, and patient satisfaction were each reported in 20% of studies. One study investigated survival using the Survival, Epidemiology, and End Results (SEER) database, observing no significant differences in overall or breast cancer-specific survival among patients receiving same-day multidisciplinary care relative to those who did not.^15^ One study^15^ reported on a structure measure by examining changes in same-day multidisciplinary care receipt over time. No studies examined balance measures.

#### Gastrointestinal cancers

Among the included MDCs for which logistics were described, 3 each operated synchronously and asynchronously. Most GI MDCs sequenced the ICR before provider visits.^21^^,^^24^^,^^26^^,^^28–31^^,^^37^ One colorectal MDC notably involved a tele-MDC whereby following an initial ICR, only the surgical specialist was physically present with the patient and the remaining providers were engaged via synchronous teleconferencing.^26^ Prior to MDC sessions, many GI clinics relied on a staff member to coordinate the completion of outstanding necessary diagnostics.^21^^,^^23^^,^^24^^,^^26^^,^^28–30^^,^^34^

Specialists from MO, RO, and surgery were involved in all GI MDCs. Among colorectal MDCs, 4 had representatives from genetics; 3 MDCs each involved nutrition, pathology, and radiology; and 2 each included gastroenterology and SW. Among hepatobiliary MDCs, all 3 had representatives from hepatology, interventional radiology, and diagnostic radiology; among pancreas MDCs, all 3 had representatives from nutrition and research. All 9 GI MDCs for which clinic frequencies were described operated on a weekly basis. MDC eligibility criteria varied across institutions, however several evaluated all patients with a new diagnosis of their respective cancer type.^23^^,^^34^^,^^37^ In hepatobiliary MDCs, patients with confirmed or suspected liver tumor^28^^,^^30^ or those with transplant-ineligible primary liver cancer^29^ were considered.

Outcome measures were the most investigated.^21^^,^^23^^,^^24^^,^^28–34^^,^^36^^,^^37^ Approximately one-fourth of studies reported on each of diagnostic interpretation changes (4/17), treatment recommendation changes (4/17), clinical trial enrollment (5/17), and survival (5/17). Conflicting findings have been reported, ranging from no significant differences to a median improvement of approximately 8 months in overall and progression-free survival among patients receiving MDC relative to non-MDC care at University of Pittsburgh Medical Center hospitals or hospital-based clinics during the same time period.^30–33^^,^^36^ Seven studies reported on process measures,^25–28^^,^^30^^,^^31^^,^^37^ among which 6 evaluated time-to-treatment with the most common definition of the first timepoint being diagnosis date. Differences in time from diagnosis to treatment initiation between MDC and non-MDC patients varied across institutions: it was significantly shorter at the Parkland Memorial liver MDC (mean, 2.3 versus 5.3 months; *P* = .002)^30^ when MDC patients were compared to pre-MDC inception historical controls and trended toward being shorter at the University of Alabama colorectal MDC (median, 30 versus 37 days; *P* = .07)^25^ when examining patients receiving care during the same time period, however no significant differences were observed at one other colorectal^27^ and pancreas MDC.^31^ Two studies reported on structure measures,^23^^,^^28^ both evaluated patient retention and one additionally patient volume. Balance measures related to resource utilization were reported by one study each on a pancreas^37^ and a hepatobiliary MDC.^28^

#### Genitourinary cancers

Most GU MDCs operated asynchronously with consecutive but separate provider visits with each relevant specialty, with the most common sequence being ICR followed by provider visits^38^^,^^39^^,^^47^^,^^50^ although two MDCs operated inversely.^41^^,^^44^ At one hospital, patients traveled to the different specialty-specific clinics located within the greater institutional complex rather than undergoing all evaluations in one physical space.^38^ Contrastingly, the one bladder MDC involved an ICR followed by a simultaneous provider visit with all relevant specialties.^39^ One center’s prostate MDC incorporated three small group sessions involving multiple patients respectively led by clinical research personnel on research opportunities, nurse educators on prostate cancer, and urologists on male sexual health.^44^

Specialists from MO, RO, and urology were involved in all GU MDCs except for two prostate MDCs which excluded MO. Six GU MDCs had representatives from research teams; four each involved pathology and radiology; and two MDCs each included genetics and SW. Three prostate MDCs were conducted weekly and another biweekly; the one bladder MDC was held weekly; the frequencies of the remaining MDCs were not described. MDC eligibility criteria for two prostate MDCs included patients with newly diagnosed cancers,^44^^,^^47^ although one additionally included cases requiring further evaluation.^47^ At the bladder MDC, all patients with muscle-invasive bladder considering therapy were evaluated.^39^

Outcome and structure measures were the most investigated GU MDC endpoints by ten^38–40^^,^^42^^,^^43^^,^^46–50^ and six^38^^,^^41^^,^^43^^,^^47–49^ of the thirteen studies, respectively. Almost one-fourth of studies reported on each of: treatment type receipt, clinical trial enrollment, patient satisfaction, and survival. Patients receiving care in MDC versus non-MDC settings were consistently reported to experience significantly improved survival,^39^^,^^40^^,^^48^ with differences as high as almost 17 months in patients at the MD Anderson Cancer Center prostate MDC as compared to a SEER-derived, propensity score-matched cohort.^40^ Patient volume and retention were reported by six and four studies, respectively, with the latter ranging from 45%^49^ to 99%.^48^ Two studies^49^^,^^50^ reported on balances measures of receipt of non-indicated imaging studies and number of physician or specialty evaluations. No studies examined process measures.

#### Head and neck cancers

Two HN MDCs operated synchronously with a simultaneous patient evaluation with all oncologic specialists^51^^,^^53^; the others did not specify whether provider visits were simultaneous or separate. When reported, all MDCs involved subsequent ICRs during which consensus recommendations were developed.

Specialists from MO, RO, and surgery were involved in all but one of the HN MDCs which excluded MO. Two HN MDCs had representatives from dentistry and speech pathology. One HN MDC each was conducted weekly and biweekly; the frequencies of the other two were not described. MDC eligibility criteria were heterogeneous across the 3 institutions in which they were described, ranging from all HN cancers^54^ to only oropharyngeal squamous cell carcinoma treated with curative intent radiation.^52^

Two studies^51^^,^^52^ have reported on MDC process measures, with conflicting findings on the association between MDC care and time-to-treatment. Similarly, two studies examined outcome measures, with one study^52^ having assessed survival differences and observing significantly improved 5-year cancer-specific survival rates among patients with stage IV disease receiving care in an MDC relative to pre-MDC inception historical controls (81% versus 54%; *P* = .009), and a trend toward improved 5-year overall survival (70% versus 51%; *P* = .072). Structure measures were reported by a single study^51^ which investigated patient retention, finding similar results between MDC to non-MDC patients. No studies examined balance measures.

#### Lung cancer

Most lung MDCs (2/3) operated asynchronously with consecutive but separate provider visits with each relevant specialty; patients at one site initially met with all relevant specialists simultaneously to discuss the overall treatment plan followed by specialty-specific visits.^56^ Each MDC facilitated an ICR immediately preceding the provider visits. Another site additionally incorporated time pre-ICR for completion of outstanding staging diagnostics and education on clinical research, nutrition, palliative/hospice care, and SW resources.^57^

Specialists from MO and RO were involved in all lung MDCs while surgery was included in 2/3. Two lung MDCs each had representatives from pulmonology and radiology. Two lung MDCs were conducted weekly; the frequency of the remaining one was not described. Eligibility criteria were described for one site which included all patients who were deemed to need interdisciplinary input.^57^

All studies^55–58^ reported on process measures, all of which evaluated and largely observed statistically significant improved time from initial oncologic assessment to treatment by approximately 10 days.^55–57^ Three studies^55^^,^^56^^,^^58^ reported on outcome measures. Survival was the most commonly evaluated outcome in 3 studies with findings ranging from no difference^58^ to a nearly 10-month improvement in overall survival among MDC relative to non-MDC patients.^55^ Two studies^56^^,^^57^ reported on balance measures which in combination observed improved rates of multidisciplinary evaluation^56^ with fewer provider visits.^57^ One study^57^ reported on a structure measure relating to incurred diagnostics-related financial costs.

#### Other cancers

Whether provider visits at other MDCs were synchronous or asynchronous was largely unspecified, however the neurologic MDC at one center involved a simultaneous evaluation with radiation oncology and neurosurgery.^62^ Most other MDCs incorporated an ICR as part of the MDC session, however two sequenced the discussion to follow the provider visits^60^^,^^61^ while one had an initial evaluation by a single oncologic provider followed by ICR and then the remaining provider visits.^64^

MO, RO, and surgery were included in 4, 3, and 3 other cancer MDCs, respectively. Other specialties commonly involved included geriatrics, nutrition, and SW. MDC eligibility criteria were reported for 2 MDCs and, although heterogeneous, both focused on evaluation of primarily older adults.^60^^,^^61^

Four studies reported on other MDC outcome measures.^59–62^ The most common outcome was patient satisfaction for which high rates were reported across two other MDCs.^59^^,^^62^ One study^64^ reported on a structure measure, observing higher total costs related to melanoma care among non-MDC relative to MDC patients. No studies reported on process or balance measures.

## Discussion

With increasingly diverse treatment options available for patients with cancer, MDCs represent a potential platform through which high-quality, patient-centered care grounded in interdisciplinary expertise may be delivered. However, we demonstrate that the implementation and reported measures of MDCs are widely heterogeneous. Deriving meaningful comparisons across studies or performing more robust systematic reviews and meta-analyses are hampered by this variability. Nevertheless, the demonstrable benefits across a range of disease diagnoses and settings underscore the importance of further investigation to better elucidate the impact of MDCs in cancer care outcomes. Notably, despite the prominence of multidisciplinary approaches in gynecologic oncology—including the management of ovarian, cervical and vulvar cancer with significant cross-disease impacts from treatment on sexual health, genetic counseling, and fertility preservation—no reports to date have specifically examined the role of MDCs in this setting.

Although we observed nearly ubiquitous involvement of MO, RO, and surgery across MDCs of all cancer diagnoses, engagement by other specialties was more varied across diagnoses and institutions. In contrast to the traditional encounter between one provider and one patient, when multiple MDC providers see a patient simultaneously within a single visit, there are necessary modifications to historical clinic structures, resources needed, and revenue sources. A prior study of various MDCs at a single comprehensive cancer center indeed found that MDCs may lack adequate resources and infrastructure relative to specialists operating in their respective specialty-specific clinics.^3^ Given the diverse staffing needs to facilitate effective interdisciplinary discussions, scheduling of MDCs and multidisciplinary conferences need to be coordinated such that specialists from MO, RO, and SO in addition to nutrition, radiology, pathology, and SW are consistently present. Further efforts are needed to optimize allocation of human resources by identifying those MDCs that most benefit from inclusion of particular specialties.

Timing of provider visits—asynchronous or synchronous—is another important aspect to consider. Relative to most breast, GU, and lung MDCs which operated asynchronously, HN MDCs were more commonly conducted synchronously. Given breast MDCs also largely evaluated those newly diagnosed, these differences may potentially reflect a greater need for upfront-only MDC planning in certain diagnoses—such as breast cancer—with less expected need for multidisciplinary input on management changes during the treatment course. In contrast, HN cancers may benefit from ongoing synchronous provider physical examinations and discussions. Given prior data suggesting different MDC models may be more appropriate for specific cancer diagnoses,^3^ further research is needed to better understand how MDCs may be optimally structured for different patient populations while also considering resource allocation efficiency, including whether multidisciplinary provider visits need to occur same-day or if similar benefits may be derived by ensuring optimal care coordination between specialty visits occurring over a narrow time period. With the growth of care networks, potential delays in time-to-treatment initiation at an institution more local to the patient following recommendations developed by the central tertiary or quaternary MDC should be considered; it may be imperative for community MO and RO to be included as part of these central MDC discussions. There is an additional need to identify potential patient-related characteristics that most benefit from MDCs. These may be both cancer-related, such as for a patient with locally-advanced rectal cancer who wants to avoid radiation to preserve fertility, or non-cancer related, such as for a patient who has limited mobility and is thus best served by a single-day visit with multiple providers.

HN and other MDCs—in contrast to GI, breast, GU, and lung—tended to operate less frequently and were structured such that ICRs followed provider visits. Decreased frequencies of MDCs for rarer cancers may allow a more efficient use of necessary resources to match local institutional demand while still meeting the complex and unique needs of patients with these cancers. Financial outcomes associated with cancer MDCs have yet to be well-elucidated but represent a needed area of investigation to further understand how MDCs may be best designed to achieve high-value care. Innovative approaches to the MDC model—including telehealth adaptations—may help to address resource constraints and make any value of multidisciplinary care more accessible to a broader reach of patients.

Limitations of this analysis included publication bias, as there are likely many more MDCs in practice than those which have been reported. Formal assessments of risk-of-bias or addressing of missing data were not performed given the narrative nature of this review. The focus on U.S. only studies was intentional; if there is transition to more episode-based payment structures in the future, the design of MDC in other countries may be more feasible to implement in the U.S.

Understanding the impact of the MDC on cancer outcomes remains a ripe area for research. Although MDCs are commonly thought to improve care outcomes, limited conflicting findings have been described regarding the presence and magnitude of potential benefits. Studies have yet to conclusively evaluate differences in cancer management plans arising from a single-visit MDC versus separate specialty-specific clinics as well as potential associated downstream impacts, such as patient comprehension of treatment options. Examination of time-to-treatment was reported by all lung MDC studies and approximately half of the GI, breast, and HN MDC studies with at least some evidence to suggest MDC relative to non-MDC patients may experience a benefit. However, whether these findings translate to clinically significant changes in outcomes—like survival or psychosocial wellbeing—remains unclear. There was a notable lack in the examined studies of impacts on patient satisfaction or patient-reported outcomes. Lastly, receipt of excess care via MDCs was not often examined and warrants further examination to ensure MDCs do not result in inappropriate overuse of care.

The impact of MDCs on addressing disparities in cancer access and outcomes will also be necessary in future studies. There will need to be intentional design to utilize and measure the specific impact of telehealth and technology on closing disparities beyond those that may exist due to patients’ geographical location.^65^ Ideally MDCs would improve care for patients who face other challenges accessing care. Whether having a single consolidated provider visit helps patients balance early cancer appointments with work and family responsibilities or longer MDC visits at more limited times make that more challenging or the quantity of information provided too overwhelming will need to be examined. Innovative solutions to provide patient-centered care that meets non-oncologic needs may align with single consolidated provider visits including childcare, in-person interpreters, palliative care and social support services. Flexibility in design, including offering hybrid telemedicine visits, providing patients with recordings of information on their treatment plans to review outside of the clinic visit, or offering MDC appointment slots at various times of the day outside of full-session clinics, may be helpful in meeting patients’ needs. Beyond measuring the impacts of these structural choices on closing disparities in care, it would be valuable to assess patient-reported outcomes, including agency in care and patient satisfaction.

## Conclusions

There has yet to be an established definition for what comprises an MDC, and it is therefore highly warranted for relevant accrediting agencies and professional organizations to develop consensus. Future investigations should clearly define MDC structures to facilitate comparisons across studies and encourage the development of new strategies for delivering multidisciplinary care.

The findings here are hypothesis-generating for the future design and prospective study of different MDC models and their potential impacts on outcomes. The most valuable synchronous MDC visit may be in those cancer types where either 1. The clinical visit, inclusive of performance status, physical exam and laboratory assessment or 2. The patient’s individual goals, may be most likely to impact the treatment plan. This is currently most applicable in tumors including head and neck, rectal, and liver cancers. In head and neck and rectal cancer, an in-office scope to directly visualize the tumor can change the treatment plan. In rectal cancer, there are curative options that omit surgery or radiation depending on the patient’s goals. In liver cancer, a patient’s performance status and laboratory tests can significantly dictate their liver-directed treatment options. In contrast, when the treatment plan can usually be defined a priori, a synchronous visit may not be as of high value for treatment decisions.

Whether the other potential value of MDCs including care coordination and patient satisfaction still make synchronous MDCs a worthwhile pursuit, is still to be determined. Future studies should prioritize measurement of standardized endpoints, including patient-reported outcomes, and cost-effectiveness to fully assess the impact that MDCs have on cancer care.

## Supplementary Material

oyag208_Supplementary_Data

## Data Availability

No new data was generated.
