# Supplementary material for: Multidisciplinary clinics in cancer—models, metrics, and meaning: a review
Source: Oncologist. 2026 Jun 4;31(7):oyag208. doi: 10.1093/oncolo/oyag208 (PMC13329076; doi:10.1093/oncolo/oyag208)
Supplement: oyag208_Supplementary_Data [file oyag208_supplementary_data.docx]

**SUPPLEMENTAL MATERIAL.** Literature search strategy.

("cancer care facilities" OR "mdc" OR "multidisciplinary care clinic*" OR "multidisciplinary cancer clinic" OR "cancer clinic" OR "multidisciplinary care team*" OR "multidisciplinary tumor board" OR "interdisciplinary communication") AND ("cancer"); Human filter
